# Prediction of $\pi$-electronic energy and physical properties of benzenoid hydrocarbons using domination degree based entropies

**DOI:** 10.1038/s41598-025-95517-6

**Published:** 2025-04-02

**Authors:** Geethu Kuriachan, A. Parthiban

**Affiliations:** Department of Mathematics, School of Advanced Sciences, Vellore Institute of Technology, Vellore, 632 014 Tamil Nadu India

**Keywords:** Domination topological indices, Domination entropies, Benzenoid hydrocarbons, Physicochemical properties, $\pi$-electronic energy, Chemistry, Mathematics and computing

## Abstract

This study introduces a novel approach to calculating graph entropies using topological indices, inspired by Shannon’s entropy concept. These entropies, as information-theoretic measures, are applied to evaluate the structural properties of chemical graphs. Graph theory is utilized to examine correlations between specific chemical properties and graph entropy measures. Within this framework, several physicochemical and quantum properties, including boiling point, enthalpy, molecular weight, and $\pi$-electronic energy are analyzed. Certain new graph entropy measures, termed domination entropies, are introduced based on domination topological indices and computed for 29 benzenoid hydrocarbons. Additionally, a QSPR analysis is conducted to investigate the linear and multilinear relationships between these entropies and the physicochemical properties, as well as the $\pi$-electronic energy of the hydrocarbons. The predictive accuracy of these new domination entropies is confirmed through various statistical tools.

## Introduction

Benzenoid hydrocarbons are a class of aromatic compounds derived from benzene, composed of fused benzene rings arranged in various configurations. These include simple structures like benzene, naphthalene, anthracene, and phenanthrene, as well as more complex polycyclic aromatic hydrocarbons (PAHs) such as pyrene, chrysene, and coronene. The structural diversity of compounds such as benzo[a]anthracene and dibenzo[a,h]anthracene arises from the different arrangements of fused rings. These hydrocarbons are notable for their stability, electronic properties, and prevalence in both natural processes and industrial applications. They are extensively studied for their roles in organic electronics, as they exhibit unique optoelectronic properties due to extended $\pi$-conjugation in larger systems like coronene and hexacene. Additionally, many of these compounds are environmental pollutants, formed as byproducts of combustion and present in fossil fuels, with implications for human health and ecological systems^1^.

Chemical graph theory, a branch of mathematical chemistry, models molecular structures using graph-theoretical methods, where atoms are vertices and bonds are edges. Topological indices, numerical values representing molecular structure, are central to this field. Originating with Wiener’s introduction of the Wiener index^2^ in 1947, which linked molecular structure to boiling points of alkanes, the field has grown significantly, driving quantitative structure-property relationship (QSPR) studies. Physicochemical properties, such as boiling point, enthalpy of vaporization, flash point, and molecular weight, represent measurable compound characteristics, while quantum-theoretic properties, such as total $\pi$-electronic energy, focus on molecular behavior at the atomic level, often calculated using Hückel’s Molecular Orbital theory. Numerous studies have explored topological indices to predict various properties of hydrocarbons. For example, Hayat et al. investigated distance-based topological descriptors for $\pi$-electronic energy in benzenoid hydrocarbons^3^, as well as distance-spectral descriptors for the same compounds^4^. Malik et al.^5^ examined degree-based topological indices for predicting the physicochemical properties of polycyclic aromatic hydrocarbons, while Hayat et al.^6^ tested spectral-based valency descriptors for these compounds. Recent studies also emphasize the predictive potential of graphical indices for both physicochemical and quantum-theoretic properties of benzenoid hydrocarbons. For instance, Sadia Noureen et al.^7^ explored the role of general multiplicative Zagreb indices in predicting the enthalpy of formation of hydrocarbons, using a dataset of 25 benzenoid hydrocarbons. Paul et al.^8^ compared multiplicative and scalar multiplicative degree-based descriptors in QSAR/QSPR studies, focusing on their effectiveness in entropy measures. Additionally, Sakander Hayat^9^ employed distance-based graphical indices to predict the thermodynamic properties of benzenoid hydrocarbons.

The notion of domination topological indices, introduced by Hanan et al.^10^, is based on domination degree in graphs, where a selected set of vertices dominates the structure of the chemical graph. These indices are valuable for understanding the structural features and connectivity patterns of chemical graphs. Domination, a pivotal concept in graph theory, plays a significant role in analyzing molecular structures, aiding in decision-making, and connecting with various graph-theoretical constructs. The concept of domination has undergone extensive exploration and development^11^. In 2021, Hanan et al.^10^ introduced various domination indices derived from minimal dominating sets, including the first Zagreb, second Zagreb, modified first Zagreb, forgotten, hyper, and modified forgotten domination indices. For additional computational studies on domination topological indices of chemical structures, readers are referred to^12–17^.

Graph entropies, rooted in Shannon’s information theory (1948)^18^, have been applied to analyze both biological and chemical systems. Rashevsky^19^ provided detailed insights into these applications, while Trucco^20^ modified Shannon’s method specifically for chemical systems. Sole^21^ and Cao^22^ studied graph entropy and introduced degree-based entropy, which can measure the heterogeneity of networks. These graph entropies have emerged as vital tools in chemical graph theory for analyzing the structural complexity and variability of molecular graphs. They quantify the distribution and arrangement of molecular features, offering insights into chemical and physical properties. Graph entropies have played a transformative role in QSPR and QSAR studies, enabling correlations between entropy measures and physicochemical properties such as boiling point, molar refractivity, and $\pi$-electronic energy. Researchers can explore additional models related to entropies in the works of^23,24^. Several studies have explored graph entropies for chemical structures, though research on benzenoid hydrocarbons using graph-theoretical entropies remains limited. Notable recent works include Konsalraj Julietraja et al.^25^ (2021), who computed analytical expressions of degree-based entropy measures for three classes of polycyclic aromatic hydrocarbons centered on a coronene molecule. In 2022, Wang et al.^26^ introduced new entropies, such as the fifth ND entropy, demonstrating its effectiveness in predicting boiling points and enthalpy, while $\pi$-electronic energy and molecular weight were best predicted using neighborhood versions of redefined Zagreb entropies. More recently, in 2023, Zuo et al.^27^ computed analytical expressions for degree-based entropy measures specific to benzenoid systems, while Hui et al.^28^ explored Ve-degree-based entropies, emphasizing their utility in correlating physicochemical and quantum-theoretic properties of 18 benzene derivatives.

Building on this literature, the present study explores the power of domination degree-based entropies for correlating physicochemical and quantum characteristics of benzenoid hydrocarbons. By incorporating additional physical properties and analyzing a larger sample size, this work introduces ten novel graph entropy measures, termed domination entropies, derived from domination topological indices, to provide deeper insights into the structural and physicochemical properties of benzenoid hydrocarbons. To facilitate this, new domination topological indices are proposed. These entropies are calculated for 29 benzenoid hydrocarbons such as “$\mathcal{B}\mathcal{H}_1$: Benzene, $\mathcal{B}\mathcal{H}_2$: Naphthalene, $\mathcal{B}\mathcal{H}_3$: Anthracene, $\mathcal{B}\mathcal{H}_4$: Phenanthrene, $\mathcal{B}\mathcal{H}_5$: Tetracene, $\mathcal{B}\mathcal{H}_6$: Benzo[c]phenanthrene, $\mathcal{B}\mathcal{H}_7$: Benzo[a]anthracene, $\mathcal{B}\mathcal{H}_8$: Chrysene, $\mathcal{B}\mathcal{H}_9$: Triphenylene, $\mathcal{B}\mathcal{H}_{10}$: Pyrene, $\mathcal{B}\mathcal{H}_{11}$: Pentacene, $\mathcal{B}\mathcal{H}_{12}$: Benzo[a]tetracene, $\mathcal{B}\mathcal{H}_{13}$: Dibenzo[a,h]anthracene, $\mathcal{B}\mathcal{H}_{14}$: Dibenzo[a,j]anthracene, $\mathcal{B}\mathcal{H}_{15}$: Pentaphene, $\mathcal{B}\mathcal{H}_{16}$: Benzo[g] chrysene, $\mathcal{B}\mathcal{H}_{17}$: Pentahelicene, $\mathcal{B}\mathcal{H}_{18}$: Benzo[c]chrysene, $\mathcal{B}\mathcal{H}_{19}$: Picene, $\mathcal{B}\mathcal{H}_{20}$: Benzo[b]chrysene, $\mathcal{B}\mathcal{H}_{21}$: Dibenzo[a,c] anthracene, $\mathcal{B}\mathcal{H}_{22}$: Dibenzo[b,g]phenanthrene, $\mathcal{B}\mathcal{H}_{23}$: Perylene, $\mathcal{B}\mathcal{H}_{24}$: Benzo[e]pyrene, $\mathcal{B}\mathcal{H}_{25}$: Benzo[a]pyrene, $\mathcal{B}\mathcal{H}_{26}$: Hexahelicene, $\mathcal{B}\mathcal{H}_{27}$: Benzo[ghi]perylene, $\mathcal{B}\mathcal{H}_{28}$: Hexacene, $\mathcal{B}\mathcal{H}_{29}$: Coronene” and their effectiveness in correlating the physicochemical properties and $\pi$-electronic energy of the hydrocarbons is thoroughly investigated.

## Domination degree based topological indices of a graph

Let $\Gamma$ be a connected simple graph with $V(\Gamma )$, a collection of vertices and $E(\Gamma )$, a collection of edges. A set $D \subset V$ is called a dominating set of $\Gamma$ if, for every vertex $x \in V$, there exists a vertex $y \in D$ such that $x$ is adjacent to $y$. A dominating set $D = \{x_1,x_2,...,x_s\}$ is minimal (M.D.S ) if $D- \{x_i\}$ is not a dominating set^16^. For every vertex $x \ \text {in} \ V (\Gamma )$, the domination degree of *x* is equal to the number of M.D.S.’s of $\Gamma$ that contain *x*, represented by $d_d(x)$^10^.

Domination degree-based topological indices (domination topological indices), including those derived from Zagreb and other indices, which are used to define the domination entropies, are outlined as follows. These indices play a crucial role in characterizing the structural properties of chemical graphs and serve as the foundation for deriving meaningful entropy measures.

- The first Zagreb domination index^10^ of $\Gamma$ denoted as $DM1(\Gamma )$, is expressed by
  1 $$\begin{aligned} DM1(\Gamma )= \sum \limits _{uv \in E(\Gamma )} d_d(u)+d_d(v) \end{aligned}$$
- The second Zagreb domination index^10^ of $\Gamma$ denoted as $DM2(\Gamma )$, is expressed by
  2 $$\begin{aligned} DM2(\Gamma )= \sum \limits _{uv \in E(\Gamma )} d_d(u) \times d_d(v) \end{aligned}$$
- The hyper Zagreb domination index^10^ of $\Gamma$ denoted as $DHM(\Gamma )$, is expressed by
  3 $$\begin{aligned} DHM(\Gamma )= \sum \limits _{uv \in E(\Gamma )} (d_d(u)+d_d(v))^2 \end{aligned}$$
- The forgetten domination index^10^ of $\Gamma$ denoted as $DF(\Gamma )$, is expressed by
  4 $$\begin{aligned} DF(\Gamma )= \sum \limits _{uv \in E(\Gamma )} (d_d(u))^2 + (d_d(v))^2 \end{aligned}$$

The following new domination topological indices are introduced here for studying various domination entropies.

- The atom-bond connectivity domination index of $\Gamma$, denoted as $DABC(\Gamma )$, is expressed by
  5 $$\begin{aligned} DABC(\Gamma )= \sum \limits _{uv \in E(\Gamma )} \sqrt{\frac{d_d(u)+d_d(v)-2}{d_d(u) \times d_d(v)}} \end{aligned}$$
- The geometric arithmetic domination index of $\Gamma$ denoted as $DGA(\Gamma )$, is expressed by
  6 $$\begin{aligned} DGA(\Gamma )= \sum \limits _{uv \in E(\Gamma )} \frac{2 \sqrt{d_d(u) \times d_d(v)}}{d_d(u)+d_d(v)} \end{aligned}$$
- The augmented Zagreb domination index of $\Gamma$ denoted as $DAZ(\Gamma )$, is expressed by
  7 $$\begin{aligned} DAZ(\Gamma )= \sum \limits _{uv \in E(\Gamma )} \bigg (\frac{d_d(u) \times d_d(v)}{d_d(u) \times d_d(v)-2}\bigg )^3 \end{aligned}$$
- The redefined first Zagreb domination index of $\Gamma$ denoted as $DM1^*(\Gamma )$, is expressed by
  8 $$\begin{aligned} DM1^*(\Gamma )= \sum \limits _{uv \in E(\Gamma )} \frac{d_d(u) + (d_d(v)}{d_d(u) \times d_d(v)} \end{aligned}$$
- The redefined second Zagreb domination index of $\Gamma$ denoted as $DM2^*(\Gamma )$, is expressed by
  9 $$\begin{aligned} DM2^*(\Gamma )= \sum \limits _{uv \in E(\Gamma )} \frac{d_d(u) \times (d_d(v)}{d_d(u) + d_d(v)} \end{aligned}$$
- The redefined third Zagreb domination index of $\Gamma$ denoted as $DM3^*(\Gamma )$, is expressed by
  10 $$\begin{aligned} DM3^*(\Gamma )= \sum \limits _{uv \in E(\Gamma )} (d_d(u) \times (d_d(v))(d_d(u) + d_d(v)) \end{aligned}$$

## Domination degree based entropies of a graph

The entropy of a graph $\Gamma$ is defined as^29^

11 $$\begin{aligned} {\mathfrak {E}}_f(\Gamma )= -\sum \limits _{i=1}^n \frac{f(v_i)}{\sum \limits _{j=1}^n f(v_j)} log \bigg ( \frac{f(v_i)}{\sum \limits _{j=1}^n f(v_j)} \bigg ), \end{aligned}$$

where *f* is an arbitrary information functional.

Let $v_i \in V$, and let $f(v_i)$ be an information function that denotes the domination degree of $v_i$, expressed as $f(v_i) = d_d(v_i)$. Consequently, Eq. (11) becomes:

12 $$\begin{aligned} {\mathfrak {E}}_f(\Gamma )&= -\sum \limits _{i=1}^n \frac{d_d(v_i)}{\sum \limits _{j=1}^n d_d(v_j)} log \bigg ( \frac{d_d(v_i)}{\sum \limits _{j=1}^n d_d(v_j)} \bigg ) \end{aligned}$$

13 $$\begin{aligned}&= log \bigg (\sum \limits _{i=1}^n d_d(v_i)\bigg ) - \frac{1}{\sum \limits _{j=1}^n d_d(v_j)} \sum \limits _{i=1}^n \bigg (d_d(v_i) log (d_d(v_i))\bigg ) \end{aligned}$$

Chen et al. introduced the entropy concept for edge-weighted graphs. Given an edge-weighted graph $\Gamma = (V(\Gamma ), E(\Gamma ), f(uv))$, where $V(\Gamma )$ is the vertex set, $E(\Gamma )$ is the edge set, and $f(uv)$ represents the weight assigned to edge $uv$, the entropy is defined as:

14 $$\begin{aligned} {\mathfrak {E}}_f(\Gamma )= -\sum \limits _{u'v'\in E(\Gamma )} \frac{f(u'v')}{\sum \limits _{uv\in E(\Gamma )} f(uv)} log \bigg ( \frac{f(u'v')}{\sum \limits _{uv\in E(\Gamma )} f(uv)} \bigg ). \end{aligned}$$

Using Eq. $14$ and considering different $f(uv)$ based on domination degree, the following new domination entropies are defined:

1. If $f(uv) = \sqrt{\frac{d_d(u)+d_d(v)-2}{d_d(u) \times d_d(v)}}$ then, by Eq.  5, $\sum \nolimits _{uv\in E(\Gamma )} f(uv) = \sum \nolimits _{uv \in E(\Gamma )} \sqrt{\frac{d_d(u)+d_d(v)-2}{d_d(u) \times d_d(v)}} = DABC(\Gamma )$. The atom-bond connectivity domination entropy is then defined by substituting the above f(uv) into Eq.  14 as follows:
  15 $$\begin{aligned} {\mathfrak {E}}_{DABC}(\Gamma ) = log (DABC(\Gamma )) - \frac{1}{DABC(\Gamma )} log \bigg [ \prod _{uv \in E(\Gamma )} \bigg (\sqrt{\frac{d_d(u)+d_d(v)-2}{d_d(u) \times d_d(v)}}\bigg )^ {\sqrt{\frac{d_d(u)+d_d(v)-2}{d_d(u) \times d_d(v)}} } \bigg ]. \end{aligned}$$
2. If, by Eq.  6, $\sum \nolimits _{uv\in E(\Gamma )} f(uv) = DGA (\Gamma )$, where $f(uv) = \frac{2 \sqrt{d_d(u) \times d_d(v)}}{d_d(u)+d_d(v)}$, then geometric arithmetic domination entropy is derived by replacing this f(uv) into Eq. 14, resulting in the following expression
  16 $$\begin{aligned} {\mathfrak {E}}_{DGA}(\Gamma ) = log (DGA(\Gamma )) - \frac{1}{DGA(\Gamma )} log \bigg [ \prod _{uv \in E(\Gamma )} \bigg (\frac{2 \sqrt{d_d(u) \times d_d(v)}}{d_d(u)+d_d(v)}\bigg )^ {\frac{2 \sqrt{d_d(u) \times d_d(v)}}{d_d(u)+d_d(v)} } \bigg ]. \end{aligned}$$
3. If, by Eq.  1, $\sum \nolimits _{uv\in E(\Gamma )} f(uv) = DM1 (\Gamma )$, where $f(uv) = d_d(u)+d_d(v)$, then the first Zagreb domination entropy is derived by replacing this f(uv) into Eq.  14, resulting in the following expression
  17 $$\begin{aligned} {\mathfrak {E}}_{DM1}(\Gamma ) = log (DM1(\Gamma )) - \frac{1}{DM1(\Gamma )} log \bigg [ \prod _{uv \in E(\Gamma )} \bigg (d_d(u)+d_d(v)\bigg )^ {d_d(u)+d_d(v)} \bigg ]. \end{aligned}$$
4. If, by Eq.  2, $\sum \nolimits _{uv\in E(\Gamma )} f(uv) = DM2 (\Gamma )$, where $f(uv) = d_d(u) \times d_d(v)$, then the second Zagreb domination entropy is derived by replacing this f(uv) into Eq.  14, resulting in the following expression
  18 $$\begin{aligned} {\mathfrak {E}}_{DM2}(\Gamma ) = log (DM2(\Gamma )) - \frac{1}{DM2(\Gamma )} log \bigg [ \prod _{uv \in E(\Gamma )} \bigg (d_d(u) \times d_d(v)\bigg )^ {d_d(u) \times d_d(v)} \bigg ]. \end{aligned}$$
5. If, by Eq.  3, $\sum \nolimits _{uv\in E(\Gamma )} f(uv) = DHM (\Gamma )$, where $f(uv) = (d_d(u) + d_d(v))^2$, then the hyper Zagreb domination entropy is derived by replacing this f(uv) into Eq.  14, resulting in the following expression
  19 $$\begin{aligned} {\mathfrak {E}}_{DHM}(\Gamma ) = log (DHM(\Gamma )) - \frac{1}{DHM(\Gamma )} log \bigg [ \prod _{uv \in E(\Gamma )} \bigg ((d_d(u) + d_d(v))^2\bigg )^ {(d_d(u) + d_d(v))^2} \bigg ]. \end{aligned}$$
6. If, by Eq.  4, $\sum \nolimits _{uv\in E(\Gamma )} f(uv) = DF (\Gamma )$, where $f(uv) = (d_d(u))^2 + (d_d(v))^2$, then the forgotten domination entropy is derived by replacing this f(uv) into Eq.  14, resulting in the following expression
  20 $$\begin{aligned} {\mathfrak {E}}_{DF}(\Gamma ) = log (DF(\Gamma )) - \frac{1}{DF(\Gamma )} log \bigg [ \prod _{uv \in E(\Gamma )} \bigg ((d_d(u))^2 + (d_d(v))^2\bigg )^ {(d_d(u))^2 + (d_d(v))^2} \bigg ]. \end{aligned}$$
7. If, by Eq.  7, $\sum \nolimits _{uv\in E(\Gamma )} f(uv) = DAZ (\Gamma )$, where $f(uv) = \bigg (\frac{d_d(u) \times d_d(v)}{d_d(u) \times d_d(v)-2}\bigg )^3$, then the Augmented Zagreb domination entropy is derived by replacing this f(uv) into Eq. 14, resulting in the following expression
  21 $$\begin{aligned} {\mathfrak {E}}_{DAZ}(\Gamma ) = log (DAZ(\Gamma )) - \frac{1}{DAZ(\Gamma )} log \bigg [ \prod _{uv \in E(\Gamma )} {\bigg (\frac{d_d(u) \times d_d(v)}{d_d(u) \times d_d(v)-2}\bigg )^3} ^{\bigg (\frac{d_d(u) \times d_d(v)}{d_d(u) \times d_d(v)-2}\bigg )^3} \bigg ]. \end{aligned}$$
8. If, by Eq.  8, $\sum \nolimits _{uv\in E(\Gamma )} f(uv) = DM1^* (\Gamma )$, where $f(uv) = \frac{d_d(u) + (d_d(v)}{d_d(u) \times d_d(v)}$, then the redefined first Zagreb domination entropy is derived by replacing this f(uv) into Eq. 14, resulting in the following expression
  22 $$\begin{aligned} {\mathfrak {E}}_{DM1^*}(\Gamma ) = log (DM1^*(\Gamma )) - \frac{1}{DM1^*(\Gamma )} log \bigg [ \prod _{uv \in E(\Gamma )} \bigg (\frac{d_d(u) + (d_d(v)}{d_d(u) \times d_d(v)}\bigg )^ {\frac{d_d(u) + (d_d(v)}{d_d(u) \times d_d(v)}} \bigg ]. \end{aligned}$$
9. If, by Eq.  9, $\sum \nolimits _{uv\in E(\Gamma )} f(uv) = DM2^* (\Gamma )$, where $f(uv) = \frac{d_d(u) \times (d_d(v)}{d_d(u) + d_d(v)}$, then the redefined second Zagreb domination entropy is derived by replacing this f(uv) into Eq. 14, resulting in the following expression
  23 $$\begin{aligned} {\mathfrak {E}}_{DM2^*}(\Gamma ) = log (DM2^*(\Gamma )) - \frac{1}{DM2^*(\Gamma )} log \bigg [ \prod _{uv \in E(\Gamma )} \bigg (\frac{d_d(u) \times (d_d(v)}{d_d(u) + d_d(v)}\bigg )^ {\frac{d_d(u) \times (d_d(v)}{d_d(u) + d_d(v)}} \bigg ]. \end{aligned}$$
10. If, by Eq.  10, $\sum \nolimits _{uv\in E(\Gamma )} f(uv) = DM3^* (\Gamma )$, where $f(uv) = (d_d(u) \times (d_d(v))(d_d(u) + d_d(v))$, then the redefined third Zagreb domination entropy is derived by replacing this f(uv) into Eq. 14, resulting in the following expression
  24 $$\begin{aligned} & {\mathfrak {E}}_{DM3^*}(\Gamma ) \nonumber \\ & \quad = log (DM3^*(\Gamma )) - \frac{1}{DM3^*(\Gamma )} log \bigg [ \prod _{uv \in E(\Gamma )} \bigg ((d_d(u) \times d_d(v))(d_d(u) + d_d(v))\bigg )^ {(d_d(u) \times d_d(v))(d_d(u) + d_d(v))} \bigg ]. \end{aligned}$$

## Method for computational analysis

This study involves two main computational tasks: evaluating domination entropies and analyzing statistical parameters. The domination entropies ${\mathfrak {E}}_{DABC}$, ${\mathfrak {E}}_{DGA}$, ${\mathfrak {E}}_{DM1}$, ${\mathfrak {E}}_{DM2}$, ${\mathfrak {E}}_{DHM}$, ${\mathfrak {E}}_{DAZ}$, ${\mathfrak {E}}_{DF}$, ${\mathfrak {E}}_{DM1^*}$, ${\mathfrak {E}}_{DM2^*}$, and ${\mathfrak {E}}_{DM3^*}$ are computed based on the domination degree of each node. Experimental data for benzenoid hydrocarbons are sourced from ChemSpider (https://www.chemspider.com/) and PubChem (https://pubchem.ncbi.nlm.nih.gov/). Regression models are developed by incorporating the computed entropies and experimental values of the hydrocarbons. Statistical analyses are performed using OriginPro software (https://www.originlab.com/), while Microsoft Excel is also suitable for regression-related calculations. The following steps outline the procedure for using regression modeling and entropy computations to determine the characteristics of benzenoid hydrocarbons:

- Compute the domination degree for each node and partition the edges of the benzenoid hydrocarbon’s molecular structures accordingly.
- Calculate the domination entropies by substituting the partitioned values into the respective entropy equations.
- Collect experimental data for the physicochemical properties of benzenoid hydrocarbons from PubChem and ChemSpider.
- Construct linear and multilinear regression models using both the computed entropies and experimental data.
- Use OriginPro software to analyze the relationship between experimental and computed values, and assess correlations between the entropies and the physicochemical properties.
- Identify domination entropies with strong correlations, as they significantly describe the properties of benzenoid hydrocarbons.

## Results

**Figure 1:**
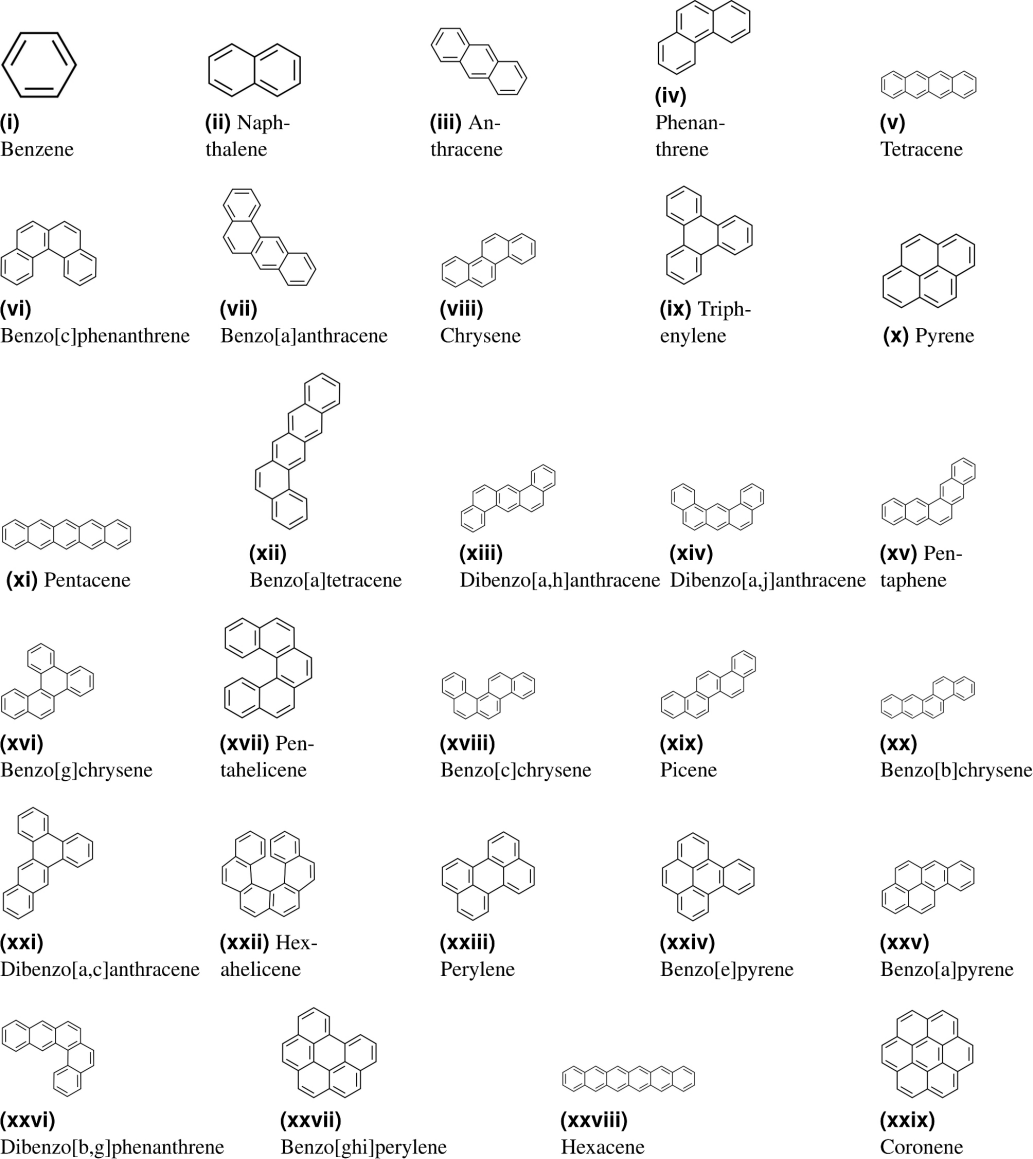
Molecular structures of benzene derivatives.

In this section, using the domination topological indices, domination entropies of 29 benzenoid hydrocarbons are evaluated. To minimize issues such as hydrogen bonding, steric effects, polar functional groups, and other factors, a family of alkanes is typically chosen as test molecules. For quality testing, 18 isomers of octane are often used to ensure the reliability of the statistical results. In contrast, statistical inferences are not applicable to benzenoid hydrocarbons like polycyclic compounds, as the chemical graphs of alkanes are acyclic. Therefore, lower benzenoid hydrocarbons have been selected as test molecules in this study, given that experimental data for the selected properties are widely available. Furthermore, no research has been conducted on domination degree-based indices for benzenoid hydrocarbons, which motivates the inclusion of these compounds in the present study. The molecular structures of 29 benzenoid hydrocarbons, sourced from the ChemSpider website, are shown in Fig. 1. It is important to note that 29 test molecules are adequate to ensure the validity of the statistical conclusions.

**Figure 2:**
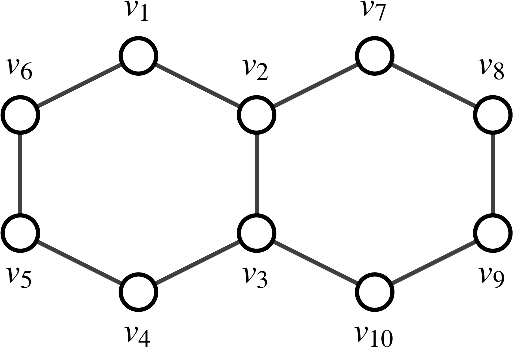
Molecular graph of naphthalene.

Consider the molecular structure of naphthalene. The molecular graph of naphthalene, depicted in Fig. 2, consists of 10 vertices and 11 edges.

Let $\Gamma$ represent this molecular graph. To determine the number of M.D.S.’s in $\Gamma$, $\Gamma$ is divided into two components, $A_1$ with vertices $\{v_1, v_2, v_3, v_4, v_5, v_6\}$, and $A_2$ with vertices $\{v_2, v_3, v_7, v_8, v_9, v_{10}\}$, and the M.D.S. for each component are calculated individually. The M.D.S.’s of $A_1$ are the following sets: $\{v_1, v_4\}, \{v_2, v_5\}, \{v_3, v_6\}, \{v_1, v_3, v_5\}, \{v_2, v_4, v_6\}.$ Thus, there are 5 M.D.S.’s for $A_1$. Similarly, the M.D.S.’s for $A_2$ are: $\{v_2, v_9\}, \{v_7, v_{10}\}, \{v_3, v_8\}, \{v_2, v_8, v_{10}\}, \{v_3, v_7, v_9\}.$ This also results in five M.D.S.’s for $A_2$. Every M.D.S. from $A_1$ is combined with each M.D.S. from $A_2$. Since there are five M.D.S.’s for $A_1$ and five M.D.S.’s for $A_2$, this results in a total of $5 \times 5 = 25$ combinations. The minimality of each combined set is verified to ensure it qualifies as a M.D.S. for $\Gamma$. Upon checking, 2 of the resulting sets are repeated, leaving a total of 23 distinct M.D.S.’s for $\Gamma$. Thus, $\Gamma$ of naphthalene has 23 minimal dominating sets and the Minimal Dominating Sets of molecular graph of naphthalene are $\{v_9, v_2, v_5\}, \{v_8, v_3, v_6\}, \{v_1, v_2, v_4, v_9\}, \{v_8, v_1, v_3, v_4\}, \{v_8, v_1, v_3, v_5\}, \{v_1, v_4, v_9, v_7\}, \{v_1, v_{10}, v_4, v_7\}, \{v_8,$ $v_1, v_4, v_9\}, \{v_8, v_1, v_{10}, v_4\}, \{v_1, v_{10}, v_5, v_7\}, \{v_8, v_1, v_{10}, v_5\}, \{v_8, v_2, v_3, v_5\}, \{v_9, v_2, v_3, v_6\}, \{v_9, v_2, v_4, v_6\},$ $\{v_2, v_{10}, v_5, v_7\}, \{v_8, v_2, v_{10}, v_5\}, \{v_9, v_3, v_6, v_7\}, \{v_{10}, v_3, v_6, v_7\}, \{v_9, v_4, v_6, v_7\}, \{v_{10}, v_4, v_6, v_7\}, \{v_{10}, v_5, v_6,$ $v_7\}, \{v_1, v_3, v_5, v_7, v_9\}, \ and \ \{v_2, v_4, v_6, v_8, v_{10}\}$.

The domination degrees of the vertices of $\Gamma$ are computed as follows:

$$\begin{aligned} & d_d(v_1) = 10, \quad d_d(v_2) = 8, \quad d_d(v_3) = 8, \quad d_d(v_4) = 10, \quad d_d(v_5) = 9, \\ & \quad d_d(v_6) = 9, \quad d_d(v_7) = 10, \quad d_d(v_8) = 9, \quad d_d(v_9) = 9, \quad d_d(v_{10}) = 10. \end{aligned}$$

The ten domination degree-based entropies are then evaluated using the computed domination degrees of the vertices of $\Gamma$.

- From Eq.  5,
  $$\begin{aligned} DABC(\Gamma )&= \sum \limits _{uv \in E(\Gamma )} \sqrt{\frac{d_d(u)+d_d(v)-2}{d_d(u) \times d_d(v)}} \\&= 4 \times \bigg (\sqrt{\frac{8+10-2}{8 \times 10}} \bigg ) + 4 \times \bigg (\sqrt{\frac{9+10-2}{9 \times 10}} \bigg ) + 2 \times \bigg (\sqrt{\frac{9+9-2}{9 \times 9}} \bigg )+ \sqrt{\frac{8+8-2}{8 \times 8}} \\&= 4.884 \end{aligned}$$
  Substituting the value of $DABC(\Gamma )$ into Eq.  15, we get:
  $$\begin{aligned} {\mathfrak {E}}_{DABC}(\Gamma )&= log (4.884) - \frac{1}{4.884} log \bigg [ \prod _{uv \in E(\Gamma )} \bigg (\sqrt{\frac{d_d(u)+d_d(v)-2}{d_d(u) \times d_d(v)}}\bigg )^ {\sqrt{\frac{d_d(u)+d_d(v)-2}{d_d(u) \times d_d(v)}} } \bigg ]\\&= 2.397 \end{aligned}$$
- From Eq.  6,
  $$\begin{aligned} DGA(\Gamma )&= \sum \limits _{uv \in E(\Gamma )} \frac{2 \sqrt{d_d(u) \times d_d(v)}}{d_d(u)+d_d(v)}\\&= 4 \times \bigg ( \frac{2 \sqrt{8 \times 10}}{8+10} \bigg ) + 4 \times \bigg (\frac{2 \sqrt{9 \times 10}}{9+10}\bigg ) + 2 \times \bigg (\frac{2 \sqrt{9 \times 9}}{9+9} \bigg ) + \frac{2 \sqrt{8 \times 8}}{8+8}\\&= 10.968 \end{aligned}$$
  Substituting the value of $DGA(\Gamma )$ into Eq.  16, we get:
  $$\begin{aligned} {\mathfrak {E}}_{DGA}(\Gamma )&= log (10.968) - \frac{1}{10.968} log \bigg [ \prod _{uv \in E(\Gamma )} \bigg (\frac{2 \sqrt{d_d(u) \times d_d(v)}}{d_d(u)+d_d(v)}\bigg )^ {\frac{2 \sqrt{d_d(u) \times d_d(v)}}{d_d(u)+d_d(v)} } \bigg ]\\&=2.3979 \end{aligned}$$
- From Eq.  1,
  $$\begin{aligned} DM1(\Gamma )&= \sum \limits _{uv \in E(\Gamma )} d_d(u)+d_d(v)\\&= 4\times (8+10)+ 4 \times (9+10)+2 \times (9+9)+1(8+8) = 200 \end{aligned}$$
  Substituting the value of $DM1(\Gamma )$ into Eq.  17, we get:
  $$\begin{aligned} {\mathfrak {E}}_{DM1}(\Gamma )&= log (200) - \frac{1}{200} log \bigg [ \prod _{uv \in E(\Gamma )} \bigg (d_d(u)+d_d(v)\bigg )^ {d_d(u)+d_d(v)} \bigg ]\\&=2.3968 \end{aligned}$$
- From Eq.  2,
  $$\begin{aligned} DM2(\Gamma )&= \sum \limits _{uv \in E(\Gamma )} d_d(u) \times d_d(v) \\&= 4 \times (8 \times 10) + 4 \times (9 \times 10) +2 \times (9 \times 9)+ (8 \times 8) = 906 \end{aligned}$$
  Substituting the value of $DM2(\Gamma )$ into Eq.  18, we get:
  $$\begin{aligned} {\mathfrak {E}}_{DM2}(\Gamma )&= log (906) - \frac{1}{906} log \bigg [ \prod _{uv \in E(\Gamma )} \bigg (d_d(u) \times d_d(v)\bigg )^ {d_d(u) \times d_d(v)} \bigg ]\\&=2.3937 \end{aligned}$$
- From Eq.  3,
  $$\begin{aligned} DHM(\Gamma )&= \sum \limits _{uv \in E(\Gamma )} (d_d(u)+d_d(v))^2 \\&= 4 \times (8+10)^2 + 4 \times (9+10)^2 + 2 \times (9+9)^2 + (8+8)^2\\&=3644 \end{aligned}$$
  Substituting the value of $DHM(\Gamma )$ into Eq.  19, we get:
  $$\begin{aligned} {\mathfrak {E}}_{DHM}(\Gamma )&= log (3644) - \frac{1}{3644} log \bigg [ \prod _{uv \in E(\Gamma )} \bigg ((d_d(u) + d_d(v))^2\bigg )^ {(d_d(u) + d_d(v))^2} \bigg ]\\&=2.3938 \end{aligned}$$
- From Eq.  4,
  $$\begin{aligned} DF(\Gamma )&= \sum \limits _{uv \in E(\Gamma )} (d_d(u))^2 + (d_d(v))^2\\&= 4 \times (8^2 + 10^2) + 4 \times (9^2 + 10^2) + 2 \times (9^2 + 9^2)+ (8^2+8^2)\\&= 1832 \end{aligned}$$
  Substituting the value of $DF(\Gamma )$ into Eq.  20, we get:
  $$\begin{aligned} {\mathfrak {E}}_{DF}(\Gamma )&= log (1832) - \frac{1}{1832} log \bigg [ \prod _{uv \in E(\Gamma )} \bigg ((d_d(u))^2 + (d_d(v))^2\bigg )^ {(d_d(u))^2 + (d_d(v))^2} \bigg ]\\&=2.3938 \end{aligned}$$
- From Eq.  7,
  $$\begin{aligned} DAZ(\Gamma )&= \sum \limits _{uv \in E(\Gamma )} \bigg (\frac{d_d(u) \times d_d(v)}{d_d(u) \times d_d(v)-2}\bigg )^3 \\&= 4 \times \bigg (\frac{8 \times 10}{8 \times 10-2}\bigg )^3 + 4 \times \bigg (\frac{9 \times 10}{9 \times 10-2}\bigg )^3 + 2 \times \bigg (\frac{9 \times 9}{9 \times 9-2}\bigg )^3 + \bigg (\frac{8 \times 8}{8 \times 8-2}\bigg )^3 \\&= 11.85 \end{aligned}$$
  Substituting the value of $DAZ(\Gamma )$ into Eq.  21, we get:
  $$\begin{aligned} {\mathfrak {E}}_{DAZ}(\Gamma )&= log (11.85) - \frac{1}{11.85} log \bigg [ \prod _{uv \in E(\Gamma )} {\bigg (\frac{d_d(u) \times d_d(v)}{d_d(u) \times d_d(v)-2}\bigg )^3} ^{\bigg (\frac{d_d(u) \times d_d(v)}{d_d(u) \times d_d(v)-2}\bigg )^3} \bigg ]\\&=2.3978 \end{aligned}$$
- From Eq.  8,
  $$\begin{aligned} DM1^*(\Gamma )&= \sum \limits _{uv \in E(\Gamma )} \frac{d_d(u) + (d_d(v)}{d_d(u) \times d_d(v)}\\&= 4\times \bigg (\frac{8 + 10}{8 \times 10}\bigg )+ 4\times \bigg (\frac{9 + 10}{9 \times 10}\bigg ) + 2 \times \bigg (\frac{9 + 9}{9 \times 9}\bigg )+ \bigg (\frac{8 + 8}{8 \times 8}\bigg )\\&=2.44 \end{aligned}$$
  Substituting the value of $DM1^*(\Gamma )$ into Eq.  22, we get:
  $$\begin{aligned} {\mathfrak {E}}_{DM1^*}(\Gamma )&= log (2.44) - \frac{1}{2.44} log \bigg [ \prod _{uv \in E(\Gamma )} \bigg (\frac{d_d(u) + (d_d(v)}{d_d(u) \times d_d(v)}\bigg )^ {\frac{d_d(u) + (d_d(v)}{d_d(u) \times d_d(v)}} \bigg ]\\&=2.3967 \end{aligned}$$
- From Eq.  9,
  $$\begin{aligned} DM2^*(\Gamma )&= \sum \limits _{uv \in E(\Gamma )} \frac{d_d(u) \times (d_d(v)}{d_d(u) + d_d(v)}\\&= 4\times \bigg (\frac{8 \times 10}{8 + 10}\bigg )+ 4\times \bigg (\frac{9 \times 10}{9 + 10}\bigg ) + 2 \times \bigg (\frac{9 \times 9}{9 + 9}\bigg )+ \bigg (\frac{8 \times 8}{8 + 8}\bigg )\\&=49.73 \end{aligned}$$
  Substituting the value of $DM2^*(\Gamma )$ into Eq.  23, we get:
  $$\begin{aligned} {\mathfrak {E}}_{DM2^*}(\Gamma )&= log (49.73) - \frac{1}{49.73} log \bigg [ \prod _{uv \in E(\Gamma )} \bigg (\frac{d_d(u) \times (d_d(v)}{d_d(u) + d_d(v)}\bigg )^ {\frac{d_d(u) \times (d_d(v)}{d_d(u) + d_d(v)}} \bigg ]\\&=2.3968 \end{aligned}$$
- From Eq.  10,
  $$\begin{aligned} DM3^*(\Gamma )&= \sum \limits _{uv \in E(\Gamma )} (d_d(u) \times (d_d(v))(d_d(u) + d_d(v))\\&= 4 \times (8 \times 10)(8+ 10) + 4 \times (9 \times 10)(9+ 10)+ \times (9 \times 9)(9+ 9) + (8 \times 8)(8+ 8)\\&=16540 \end{aligned}$$
  Substituting the value of $DM3^*(\Gamma )$ into Eq.  24, we get:
  $$\begin{aligned} {\mathfrak {E}}_{DM3^*}(\Gamma )&= log (16540) - \\ &\frac{1}{16540} log \bigg [ \prod _{uv \in E(\Gamma )} \bigg ((d_d(u) \times d_d(v))(d_d(u) + d_d(v))\bigg )^ {(d_d(u) \times d_d(v))(d_d(u) + d_d(v))} \bigg ]\\&=2.3889 \end{aligned}$$

Similarly, the entropies of the remaining 28 benzenoid hydrocarbons are calculated and verified using validation method in Python. The computed values are presented in Table 1.

## QSPR analysis of physicochemical properties and $\pi$-electronic energy of benzenoid hydrocarbons

This section explores the QSPR analysis of physicochemical properties and $\pi$-electronic energy of benzenoid hydrocarbons using domination degree-based entropies. The analysis is based on experimental data from PubChem and reference^3^. The dataset includes properties such as boiling point (BP), LogP, molar refractivity (MR), enthalpy of vaporization (E), flash point (FP), polarizability (P), molecular weight (MW), XLogP3, complexity (C), and $\pi$-electronic energy (PE). Table 1 presents the entropy values for benzenoid hydrocarbons, while Table 4 lists the numerical values for these physicochemical properties and $\pi$-electronic energy. OriginPro software is used to develop linear and multilinear regression models for predicting the physicochemical properties and $\pi$-electronic energy of benzenoid hydrocarbons.

**Table 1:** Calculated values of domination degree-based entropies.

Molecule	${\mathfrak {E}}_{DABC}$	${\mathfrak {E}}_{DGA}$	${\mathfrak {E}}_{DM1}$	${\mathfrak {E}}_{DM2}$	${\mathfrak {E}}_{DHM}$	${\mathfrak {E}}_{DAZ}$	${\mathfrak {E}}_{DF}$	${\mathfrak {E}}_{DM1^*}$	${\mathfrak {E}}_{DM2^*}$	${\mathfrak {E}}_{DM3^*}$
$\mathcal{B}\mathcal{H}_1$	1.7917	1.7917	1.7917	1.7917	1.7917	1.7917	1.7917	1.7917	1.7917	1.7917
$\mathcal{B}\mathcal{H}_2$	2.3977	2.3979	2.3968	2.3937	2.3938	2.3978	2.3938	2.3967	2.3968	2.3889
$\mathcal{B}\mathcal{H}_3$	2.7722	2.7726	2.7710	2.7666	2.7666	2.7726	2.7666	2.7709	2.7710	2.7598
$\mathcal{B}\mathcal{H}_4$	2.7723	2.7726	2.7716	2.7696	2.7686	2.7726	2.7686	2.7716	2.7716	2.7638
$\mathcal{B}\mathcal{H}_5$	3.0442	3.0445	3.0433	3.0400	3.0401	3.0445	3.0401	3.0432	3.0433	3.0351
$\mathcal{B}\mathcal{H}_6$	3.0440	3.0445	3.0428	3.0375	3.0378	3.0445	3.0381	3.0425	3.0426	3.0294
$\mathcal{B}\mathcal{H}_7$	3.0443	3.0445	3.0436	3.0409	3.0409	3.0445	3.0408	3.0436	3.0436	3.0365
$\mathcal{B}\mathcal{H}_8$	3.0442	3.0445	3.0431	3.0392	3.0391	3.0445	3.0389	3.0431	3.0432	3.0327
$\mathcal{B}\mathcal{H}_9$	3.0439	3.0445	3.0419	3.0343	3.0342	3.0445	3.0342	3.042	3.0419	3.0214
$\mathcal{B}\mathcal{H}_{10}$	2.9439	2.9444	2.9426	2.9375	2.9375	2.9444	2.9375	2.9425	2.9426	2.9295
$\mathcal{B}\mathcal{H}_{11}$	3.2578	3.2581	3.2575	3.2556	3.2556	3.2581	3.2539	3.2569	3.2570	3.2491
$\mathcal{B}\mathcal{H}_{12}$	3.2579	3.2581	3.2570	3.2539	3.2539	3.2581	3.2559	3.2574	3.2575	3.2527
$\mathcal{B}\mathcal{H}_{13}$	3.2579	3.2581	3.2573	3.2551	3.2552	3.2581	3.2552	3.2573	3.2573	3.2515
$\mathcal{B}\mathcal{H}_{14}$	3.2578	3.2581	3.2571	3.2543	3.2544	3.2581	3.2544	3.2571	3.2571	3.2497
$\mathcal{B}\mathcal{H}_{15}$	3.2579	3.2581	3.2571	3.2543	3.2542	3.2581	3.2541	3.2571	3.2571	3.2495
$\mathcal{B}\mathcal{H}_{16}$	3.2574	3.2581	3.2556	3.2482	3.2483	3.2581	3.2485	3.2555	3.2555	3.2362
$\mathcal{B}\mathcal{H}_{17}$	3.2577	3.2581	3.2566	3.2521	3.2521	3.2581	3.2521	3.2565	3.2566	3.2451
$\mathcal{B}\mathcal{H}_{18}$	3.2577	3.2581	3.2566	3.2523	3.2522	3.2581	3.2522	3.2566	3.2566	3.2452
$\mathcal{B}\mathcal{H}_{19}$	3.2578	3.2581	3.2569	3.2536	3.2537	3.2581	3.2536	3.2569	3.2569	3.2482
$\mathcal{B}\mathcal{H}_{20}$	3.2578	3.2581	3.2563	3.2536	3.2535	3.2581	3.2533	3.2569	3.2569	3.2481
$\mathcal{B}\mathcal{H}_{21}$	3.2576	3.2581	3.2569	3.2511	3.2511	3.2581	3.2509	3.2563	3.2563	3.2425
$\mathcal{B}\mathcal{H}_{22}$	3.2577	3.2581	3.2566	3.2515	3.2522	3.2581	3.2522	3.2565	3.2565	3.2451
$\mathcal{B}\mathcal{H}_{23}$	3.1771	3.1780	3.1753	3.1659	3.1674	3.1780	3.1687	3.1739	3.1744	3.1533
$\mathcal{B}\mathcal{H}_{24}$	3.1774	3.1780	3.1756	3.1683	3.1685	3.1780	3.1687	3.1754	3.1755	3.1568
$\mathcal{B}\mathcal{H}_{25}$	3.1775	3.1780	3.1759	3.1699	3.1699	3.1780	3.1699	3.1759	3.1759	3.1602
$\mathcal{B}\mathcal{H}_{26}$	3.4335	3.4339	3.4322	3.4269	3.4272	3.4339	3.4275	3.4319	3.4321	3.4188
$\mathcal{B}\mathcal{H}_{27}$	3.2952	3.2958	3.3281	3.2871	3.2873	3.2958	3.2876	3.2934	3.2934	3.2781
$\mathcal{B}\mathcal{H}_{28}$	3.4338	3.4339	3.4335	3.4321	3.4322	3.4359	3.4321	3.4335	3.4335	3.4299
$\mathcal{B}\mathcal{H}_{29}$	3.4006	3.4012	3.3990	3.3928	3.3928	3.4012	3.3929	3.3988	3.3990	3.3829

**Figure 3:**
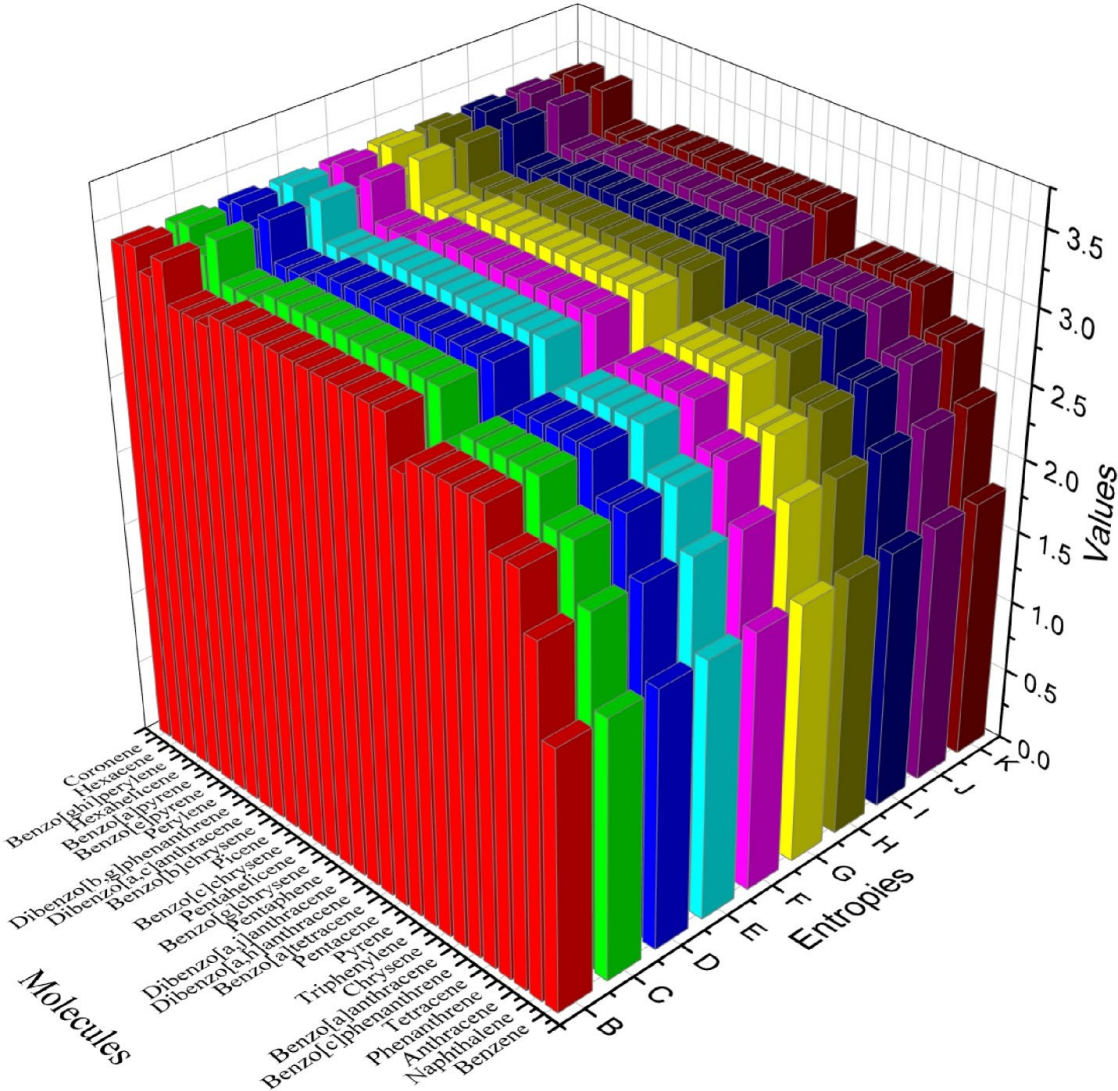
Graphical representation of the entropies of the 29 benzenoid hydrocarbons.

The graphical comparison of the entropies of the 29 benzenoid hydrocarbons is depicted in Fig. 3. This figure provides a clear visual representation of the variations in entropy across the different hydrocarbons, highlighting trends and patterns. This comparison serves as a useful tool for understanding the relationship between molecular structure and entropy in these compounds. In this figure, B, C, D, E, F, G, H, I, J, and K represent ${\mathfrak {E}}_{DABC}$, ${\mathfrak {E}}_{DGA}$, ${\mathfrak {E}}_{DM1}$, ${\mathfrak {E}}_{DM2}$, ${\mathfrak {E}}_{DHM}$, ${\mathfrak {E}}_{DAZ}$, ${\mathfrak {E}}_{DF}$, ${\mathfrak {E}}_{DM1^*}$, ${\mathfrak {E}}_{DM2^*}$, and ${\mathfrak {E}}_{DM3^*}$, respectively.

**Table 2:** Physicochemical properties and $\pi$-electronic energy of benzenoid hydrocarbons.

Molecule	BP	LogP	MR	E	FP	P	MW	XLogP3	C	PE
$\mathcal{B}\mathcal{H}_1$	80.1	2.03	25.28	30.7	-11.1	10.4	78.11	2.1	15.5	8
$\mathcal{B}\mathcal{H}_2$	218	3.03	42.45	43.9	78.9	17.5	128.17	3.3	80.6	13.6832
$\mathcal{B}\mathcal{H}_3$	340	4.03	59.62	55.8	146.6	24.6	178.23	4.4	154	19.3137
$\mathcal{B}\mathcal{H}_4$	340	4.03	59.62	55.8	146.6	24.6	178.23	4.5	174	19.4483
$\mathcal{B}\mathcal{H}_5$	432.02	5.9	76.79	66.7	209.1	31.6	228.3	5.9	236	24.9308
$\mathcal{B}\mathcal{H}_6$	436.7	5.03	76.79	66.7	209.1	31.6	228.3	5.7	266	25.1875
$\mathcal{B}\mathcal{H}_7$	438	5.03	76.79	66.7	209.1	31.6	228.3	5.8	294	25.1012
$\mathcal{B}\mathcal{H}_8$	448	5.03	76.79	67.9	209.1	31.6	228.3	5.7	264	25.1922
$\mathcal{B}\mathcal{H}_9$	438	5.03	76.79	65.3	209.1	31.6	228.3	4.9	217	25.2745
$\mathcal{B}\mathcal{H}_{10}$	404	5.08	68.36	63	168.8	28.7	202.25	4.9	217	22.5055
$\mathcal{B}\mathcal{H}_{11}$	524.6	6.02	93.96	76.9	264.5	38.7	278.3	6.7	325	30.544
$\mathcal{B}\mathcal{H}_{12}$	547.5	6.02	93.96	76.9	264.5	38.7	278.3	6.7	399	30.7255
$\mathcal{B}\mathcal{H}_{13}$	524	6.02	93.96	76.9	264.5	38.7	278.3	6.5	361	30.8805
$\mathcal{B}\mathcal{H}_{14}$	524.7	6.02	93.96	76.9	264.5	38.7	278.3	6.5	363	30.8795
$\mathcal{B}\mathcal{H}_{15}$	547.5	6.02	93.96	76.9	264.5	38.7	278.3	6.7	361	30.7627
$\mathcal{B}\mathcal{H}_{16}$	525	6.02	93.96	76.9	264.5	38.7	278.3	7	399	30.999
$\mathcal{B}\mathcal{H}_{17}$	524.7	6.02	93.96	76.9	264.5	38.7	278.3	7	361	30.9362
$\mathcal{B}\mathcal{H}_{18}$	524.7	6.02	93.96	76.9	264.5	38.7	278.3	7	399	30.9386
$\mathcal{B}\mathcal{H}_{19}$	520	6.02	93.96	76.2	264.5	38.7	278.3	7	361	30.9432
$\mathcal{B}\mathcal{H}_{20}$	524.7	6.02	93.96	76.9	264.5	38.7	278.3	6.5	399	30.839
$\mathcal{B}\mathcal{H}_{21}$	518	6.02	93.96	76.1	264.5	38.7	278.3	6.7	361	30.9418
$\mathcal{B}\mathcal{H}_{22}$	524.7	6.02	93.96	76.1	264.5	38.7	278.3	6.5	399	30.8336
$\mathcal{B}\mathcal{H}_{23}$	400	5.34	85.53	70.2	228.6	35.8	252.3	5.8	304	28.2453
$\mathcal{B}\mathcal{H}_{24}$	467.5	5.34	85.53	70.2	228.6	35.8	252.3	6.4	336	28.3361
$\mathcal{B}\mathcal{H}_{25}$	495	5.34	85.53	73.4	228.6	35.8	252.3	6	372	28.222
$\mathcal{B}\mathcal{H}_{26}$	604.1	7.45	115.5	86.5	314.6	45.8	328.4	7.9	464	36.6814
$\mathcal{B}\mathcal{H}_{27}$	500	5.66	94.28	74.1	247.2	40	328.4	6.6	411	31.4251
$\mathcal{B}\mathcal{H}_{28}$	604	7.02	111.13	86.5	314.6	45.8	328.4	8.2	418	36.1557
$\mathcal{B}\mathcal{H}_{29}$	525	5.98	103.02	77	265.2	44.1	300.4	7.2	376	34.5718

### Linear regression model

The linear regression model^30^ utilized in this study is given by:

$$\begin{aligned} P = \alpha + \beta {\mathfrak {E}} \end{aligned}$$

where $P$ represents a physicochemical property or $\pi$-electronic energy, ${\mathfrak {E}}$ denotes the entropy, and $\alpha$ and $\beta$ are the regression coefficients. This model is used to establish a linear relationship between the property $P$ and the corresponding entropy ${\mathfrak {E}}$. For each regression model, the correlation coefficient r, standard error (SE), F-test value, and probability (Prob) are computed to evaluate the model’s fit. A correlation coefficient r$\ge$ 0.8 across all models indicates statistical significance, validating the reliability of the results.

#### Discussion

Tables 3, 4, and 5 present regression models and their statistical parameters for the physicochemical properties and $\pi$-electronic energy of benzenoid hydrocarbons. Table 3 uses ${\mathfrak {E}}_{DABC}$, ${\mathfrak {E}}_{DGA}$, ${\mathfrak {E}}_{DM1}$, and ${\mathfrak {E}}_{DM2}$, while Table 4 includes ${\mathfrak {E}}_{DHM}$, ${\mathfrak {E}}_{DF}$, ${\mathfrak {E}}_{DAZ}$, and ${\mathfrak {E}}_{DM1^*}$. Table 5 focuses on ${\mathfrak {E}}_{DM2^*}$ and ${\mathfrak {E}}_{DM3^*}$.

**Table 3:** Statistical analysis of physical properties and PE using ${\mathfrak {E}}_{DABC}$, ${\mathfrak {E}}_{DGA}$, ${\mathfrak {E}}_{DM1}$, and ${\mathfrak {E}}_{DM2}$.

Regression equation	r	SE	F	Prob
PE = 18. 33285 ${\mathfrak {E}}_{DABC}$ - 29.227739	0.97009	1.56103	431.1685	$<0.0001$
BP = 322.85032 ${\mathfrak {E}}_{DABC}$ - 537.37708	0.97286	26.1292	477.2659	$<0.0001$
Log P = 3.09439 ${\mathfrak {E}}_{DABC}$ - 4.14779	0.94732	0.35601	236.1735	$<0.0001$
MR = 55.29543 ${\mathfrak {E}}_{DABC}$ - 87.38109	0.96537	5.08438	369.7557	$<0.0001$
E = 34.52117 ${\mathfrak {E}}_{DABC}$ - 36.86177	0.97783	2.51563	588.6933	$<0.0001$
FP = 200.75264 ${\mathfrak {E}}_{DABC}$ - 396.8415	0.98204	13.1224	731.6472	$<0.0001$
P = 22.82567 ${\mathfrak {E}}_{DABC}$ - 36.03395	0.96973	1.95572	425.8377	$<0.0001$
MW = 162.38246 ${\mathfrak {E}}_{DABC}$ - 253.0992	0.95766	16.6101	298.7747	$<0.0001$
XLog = 3.68957 ${\mathfrak {E}}_{DABC}$ - 5.38899	0.94935	0.41551	246.4859	$<0.0001$
C = 296.12223 ${\mathfrak {E}}_{DABC}$ - 606.535	0.92689	40.8031	164.6522	$<0.0001$
PE = 18.32822 ${\mathfrak {E}}_{DGA}$ - 29.27008	0.97005	1.56211	430.5385	$<0.0001$
BP = 322.751944 ${\mathfrak {E}}_{DGA}$ - 537.19612	0.97277	26.1732	475.5756	$<0.0001$
Log P = 3.09346 ${\mathfrak {E}}_{DGA}$ - 4.14211	0.94723	0.35629	235.7643	$<0.0001$
MR = 55.28073 ${\mathfrak {E}}_{DGA}$ - 87.35678	0.96532	5.08829	369.8907	$<0.0001$
E = 34.5116 ${\mathfrak {E}}_{DGA}$ - 36.84539	0.97776	2.51933	586.8870	$<0.0001$
FP = 200.69822 ${\mathfrak {E}}_{DGA}$ - 396.74993	0.98198	13.1443	729.1271	$<0.0001$
P = 22.81995 ${\mathfrak {E}}_{DGA}$ - 36.025	0.96969	1.95698	425.2540	$<0.0001$
MW = 162.34168 ${\mathfrak {E}}_{DGA}$ - 253.03523	0.95766	16.6101	298.7747	$<0.0001$
XLog = 3.68847 ${\mathfrak {E}}_{DGA}$ - 5.387	0.94927	0.41585	246.0455	$<0.0001$
C = 296.04406 ${\mathfrak {E}}_{DGA}$ - 606.4065	0.92684	40.8166	164.5252	$<0.0001$
PE = 18.30879 ${\mathfrak {E}}_{DM1}$ - 29.0423	0.97015	3.63234	432.0720	$<0.0001$
BP = 321.99262 ${\mathfrak {E}}_{DM1}$ - 534.746	0.97199	26.5401	461.7725	$<0.0001$
Log P = 3.08477 ${\mathfrak {E}}_{DM1}$ - 4.11424	0.94604	0.36018	230.1122	$<0.0001$
MR = 55.19059 ${\mathfrak {E}}_{DM1}$ - 87.06113	0.96524	5.09376	368.2947	$<0.0001$
E = 34.4294 ${\mathfrak {E}}_{DM1}$ - 36.58026	0.97695	2.56443	565.4842	$<0.0001$
FP = 200.20266 ${\mathfrak {E}}_{DM1}$ - 395.15434	0.98108	13.4659	693.4372	$<0.0001$
P = 22.79318 ${\mathfrak {E}}_{DM1}$ - 35.93538	0.97006	1.94525	430.7261	$<0.0001$
MW = 162.59767 ${\mathfrak {E}}_{DM1}$ - 253.78458	0.96063	16.0308	322.7449	$<0.0001$
XLog = 3.68147 ${\mathfrak {E}}_{DM1}$ - 5.36421	0.94894	0.41716	246.3310	$<0.0001$
C = 296.0351 ${\mathfrak {E}}_{DM1}$ - 606.295	0.92825	40.4355	168.1527	$<0.0001$
PE = 18.3935 ${\mathfrak {E}}_{DM2}$ - 29.36633	0.9706	1.54791	438.9692	$<0.0001$
BP = 324.1008 ${\mathfrak {E}}_{DM2}$ - 539.50834	0.97392	25.6220	497.4284	$<0.0001$
Log P = 3.10643 ${\mathfrak {E}}_{DM2}$ - 4.16438	0.94836	0.35256	241.3602	$<0.0001$
MR = 55.48686 ${\mathfrak {E}}_{DM2}$ - 87.67555	0.96603	5.03704	377.2487	$<0.0001$
E = 34.64625 ${\mathfrak {E}}_{DM2}$ - 37.06288	0.97865	2.46925	612.0398	$<0.0001$
FP = 201.46202 ${\mathfrak {E}}_{DM2}$ - 397.95512	0.98278	12.8536	763.7119	$<0.0001$
P = 22.9009 ${\mathfrak {E}}_{DM2}$ - 36.14384	0.97023	1.93985	433.2776	$<0.0001$
MW = 162.91867 ${\mathfrak {E}}_{DM2}$ - 253.88348	0.95816	16.5148	302.5478	$<0.0001$
XLog = 3.70411 ${\mathfrak {E}}_{DM2}$ - 5.41412	0.95045	0.41111	252.3780	$<0.0001$
C = 297.10472 ${\mathfrak {E}}_{DM2}$ - 607.9796	0.92738	40.6703	165.9062	$<0.0001$

**Table 4:** Statistical analysis of physical properties and PE using ${\mathfrak {E}}_{DHM}$, ${\mathfrak {E}}_{DF}$, ${\mathfrak {E}}_{DAZ}$, and ${\mathfrak {E}}_{DM1^*}$.

Regression equation	r	SE	F	Prob
PE = 18.39038 ${\mathfrak {E}}_{DHM}$ - 29.35813	0.97063	1.54694	439.5535	$<0.0001$
BP = 323.9987 ${\mathfrak {E}}_{DHM}$ - 539.21728	0.97381	26.6727	495.3607	$<0.0001$
Log P = 3.10584 ${\mathfrak {E}}_{DHM}$ - 4.16281	0.94838	0.35249	241.4533	$<0.0001$
MR = 55.47739 ${\mathfrak {E}}_{DHM}$ - 87.65078	0.96606	5.03434	377.6817	$<0.0001$
E = 34.639108 ${\mathfrak {E}}_{DHM}$ - 37.0435	0.97865	2.46915	612.0926	$<0.0001$
FP = 200.42106 ${\mathfrak {E}}_{DHM}$ - 397.84478	0.98278	12.8516	763.9609	$<0.0001$
P = 22.89712 ${\mathfrak {E}}_{DHM}$ - 36.13394	0.97027	1.93852	433.9097	$<0.0001$
MW = 162.89102 ${\mathfrak {E}}_{DHM}$ - 253.81121	0.95820	16.5075	302.8371	$<0.0001$
XLog = 3.70328 ${\mathfrak {E}}_{DHM}$ - 5.41184	0.95044	0.41117	252.2921	$<0.0001$
C = 297.05635 ${\mathfrak {E}}_{DHM}$ - 607.85418	0.92742	40.6587	166.0155	$<0.0001$
PE = 18.38906 ${\mathfrak {E}}_{DF}$ - 29.3553	0.97064	1.54685	439.6083	$<0.0001$
BP = 323.94828 ${\mathfrak {E}}_{DF}$ - 539.0843	0.97374	25.7099	493.8480	$<0.0001$
Log P = 3.10547 ${\mathfrak {E}}_{DF}$ - 4.16187	0.94834	0.35263	241.2405	$<0.0001$
MR = 55.47295 ${\mathfrak {E}}_{DF}$ - 87.64085	0.96606	5.03466	377.6302	$<0.0001$
E = 34.63516 ${\mathfrak {E}}_{DF}$ - 37.0375	0.97861	2.47125	611.0052	$<0.0001$
FP = 201.39918 ${\mathfrak {E}}_{DF}$ - 397.79083	0.98275	12.8637	762.4791	$<0.0001$
P = 22.89549 ${\mathfrak {E}}_{DF}$ - 36.13044	0.97027	1.93840	433.9685	$<0.0001$
MW = 162.88001 ${\mathfrak {E}}_{DF}$ - 253.7883	0.95820	16.5061	302.8952	$<0.0001$
XLog = 3.70282 ${\mathfrak {E}}_{DF}$ - 5.41068	0.95039	0.41136	252.0414	$<0.0001$
C = 297.07348 ${\mathfrak {E}}_{DF}$ - 607.9278	0.92755	40.6255	166.3319	$<0.0001$
PE = 18.32557 ${\mathfrak {E}}_{DAZ}$ - 29.26306	0.97013	1.55992	431.8255	$<0.0001$
BP = 322.6983 ${\mathfrak {E}}_{DAZ}$ - 537.0568	0.97728	26.1425	476.7545	$<0.0001$
Log P = 3.09309 ${\mathfrak {E}}_{DAZ}$ - 4.14117	0.94734	0.35500	236.2859	$<0.0001$
MR = 55.27347 ${\mathfrak {E}}_{DAZ}$ - 87.33785	0.96542	5.08125	370.2443	$<0.0001$
E = 34.50672 ${\mathfrak {E}}_{DAZ}$ - 36.83247	0.97785	2.51435	589.3214	$<0.0001$
FP = 200.6658 ${\mathfrak {E}}_{DAZ}$ - 396.6624	0.98205	13.1191	732.0350	$<0.0001$
P = 22.81688 ${\mathfrak {E}}_{DAZ}$ - 36.01697	0.96979	1.95396	426.6566	$<0.0001$
MW = 162.31944 ${\mathfrak {E}}_{DAZF}$ - 252.9767	0.95772	16.6002	299.1634	$<0.0001$
XLog = 3.68821 ${\mathfrak {E}}_{DAZ}$ - 5.38642	0.94942	0.41524	246.8464	$<0.0001$
C = 295.98082 ${\mathfrak {E}}_{DAZI}$ - 606.2294	0.92685	40.8119	166.5690	$<0.0001$
PE = 18.34581 ${\mathfrak {E}}_{DM1^*}$ - 29.29558	0.97022	1.55770	433.1303	$<0.0001$
BP = 323.14123 ${\mathfrak {E}}_{DM1^*}$ - 537.8919	0.97318	25.9775	483.1739	$<0.0001$
Log P = 3.097 ${\mathfrak {E}}_{DM1^*}$ - 4.14817	0.94757	0.35517	237.4223	$<0.0001$
MR = 55.33622 ${\mathfrak {E}}_{DM1^*}$ - 87.44122	0.96553	5.07292	371.5496	$<0.0001$
E = 34.54835 ${\mathfrak {E}}_{DM1^*}$ - 36.90464	0.97780	2.50386	594.4935	$<0.0001$
FP = 200.90644 ${\mathfrak {E}}_{DM1^*}$ - 397.07762	0.98223	13.0537	739.6621	$<0.0001$
P = 22.8416 ${\mathfrak {E}}_{DM1^*}$ - 36.05597	0.96985	1.95187	427.6272	$<0.0001$
MW = 162.49736 ${\mathfrak {E}}_{DM1^*}$ - 253.606	0.95779	16.5855	299.7492	$<0.0001$
XLog = 3.69273 ${\mathfrak {E}}_{DM1^*}$ - 5.39437	0.94962	0.41440	247.9086	$<0.0001$
C = 296.3491 ${\mathfrak {E}}_{DM1^*}$ - 606.88313	0.92707	40.7551	165.1037	$<0.0001$

**Table 5:** Statistical analysis of physical properties and PE using ${\mathfrak {E}}_{DM2^*}$ and ${\mathfrak {E}}_{DM3^*}.$.

Regression equation	r	SE	F	Prob
EP = 18. 34517 ${\mathfrak {E}}_{DM2^*}$ - 29.29478	0.97021	1.5578	433.0704	$<0.0001$
BP = 322.11534 ${\mathfrak {E}}_{DM2^*}$ - 537.83266	0.97313	26.0003	482.2793	$<0.0001$
Log P = 3.09686 ${\mathfrak {E}}_{DM2^*}$ - 4.14795	0.94756	0.35521	237.3639	$<0.0001$
MR = 55.33429 ${\mathfrak {E}}_{DM2^*}$ - 87.43885	0.96553	5.07319	371.5071	$<0.0001$
E = 34.54668 ${\mathfrak {E}}_{DM2^*}$ - 36.90169	0.97802	2.50483	594.0134	$<0.0001$
FP = 200.89643 ${\mathfrak {E}}_{DM2^*}$ - 397.05955	0.98221	13.0605	738.8643	$<0.0001$
P = 22.84089 ${\mathfrak {E}}_{DM2^*}$ - 36.05526	0.96985	1.95187	427.6267	$<0.0001$
MW = 162.49126 ${\mathfrak {E}}_{DM2^*}$ - 253.2523	0.95779	16.5866	299.6979	$<0.0001$
XLog = 3.69258 ${\mathfrak {E}}_{DM2^*}$ - 5.39412	0.94961	0.41448	247.8492	$<0.0001$
C = 296.3332 ${\mathfrak {E}}_{DM2^*}$ - 606.8532	0.92704	40.7607	165.0507	$<0.0001$
EP = 18. 46545 ${\mathfrak {E}}_{DM3^*}$ - 29.46269	0.97125	1.53085	449.4168	$<0.0001$
BP = 325.56903 ${\mathfrak {E}}_{DM3^*}$ - 541.82705	0.97518	25.0047	523.6441	$<0.0001$
Log P = 3.12093 ${\mathfrak {E}}_{DM3^*}$ - 4.18793	0.94972	0.34802	248.4016	$<0.0001$
MR = 55.71429 ${\mathfrak {E}}_{DM3^*}$ - 87.9985	0.96686	4.97601	387.2257	$<0.0001$
E = 34.79381 ${\mathfrak {E}}_{DM3^*}$ - 37.28171	0.97965	2.41138	643.0819	$<0.0001$
FP = 202.29477 ${\mathfrak {E}}_{DM3^*}$ - 399.14936	0.98366	12.5235	805.9598	$<0.0001$
P = 22.99017 ${\mathfrak {E}}_{DM3^*}$ - 36.26282	0.97087	1.91919	443.2443	$<0.0001$
MW = 163.55374 ${\mathfrak {E}}_{DM3^*}$ - 254.73053	0.95879	16.3918	307.5080	$<0.0001$
XLog = 3.72135 ${\mathfrak {E}}_{DM3^*}$ - 5.44205	0.95180	0.40563	259.9772	$<0.0001$
C = 296.36913 ${\mathfrak {E}}_{DM3^*}$ - 609.85315	0.92833	40.4194	168.3514	$<0.0001$

From the data in Tables 3, 4, and 5, the following observations can be made:

- The analysis demonstrates that the model estimating the effect of entropies ${\mathfrak {E}}_{DM3^*}$ on PE exhibits the strongest statistical metrics, with a high correlation coefficient (r=0.97125), an F-value of 449.416, the lowest standard error (SE=1.5308), and a probability of $<0.0001$. These indicators confirm the model’s robustness, highlighting ${\mathfrak {E}}_{DM3^*}$ as the most significant predictors of PE. Nevertheless, the other nine models also show strong statistical parameters, with values close to those of the top model. Thus, other entropies also serve as good predictors of PE.
- The findings reveal that the model predicting BP based on ${\mathfrak {E}}_{DM3^*}$ achieves the highest values for both the coefficient of determination (r=0.97518) and the F-value (F=523.644), along with the lowest standard error (SE=25.0047). This underscores the model’s high significance and indicates that ${\mathfrak {E}}_{DM3^*}$ has a substantial influence on BP, making it the strongest predictor of BP. Similarly, models incorporating other entropies also demonstrate robust statistical metrics, providing good predictive performance for BP.
- The results indicate that the estimated model for LogP based on ${\mathfrak {E}}_{DM3^*}$ achieves the highest r and F-values (r=0.9497, F=248.4016), along with the lowest standard error (SE=0.34802). This demonstrates that the model is highly significant and that ${\mathfrak {E}}_{DM3^*}$ has a substantial impact on LogP. Consequently, ${\mathfrak {E}}_{DM3^*}$ is the most effective predictor of LogP. Additionally, entropies ${\mathfrak {E}}_{DM2}$, ${\mathfrak {E}}_{DHM}$, and ${\mathfrak {E}}_{DF}$ also perform well in predicting LogP.
- The results demonstrate that the model for MR based on ${\mathfrak {E}}_{DM3^*}$ exhibits the highest r and F-values (r=0.9668, F=387.2257), along with the lowest standard error (SE=4.976). This suggests that ${\mathfrak {E}}_{DM3^*}$ significantly impacts MR, making it the most powerful predictor of MR. Additionally, other entropies also produce notable results.
- The E can be reliably predicted using all entropy indices, as each model’s r-value falls between 0.976 and 0.979. Based on the statistical parameters of these regression models, all models are significant for predicting E. Among them, ${\mathfrak {E}}_{DM3^*}$ demonstrated particularly strong significance.
- The findings show that the model for FP using ${\mathfrak {E}}_{DM3^*}$, ${\mathfrak {E}}_{DM2}$, ${\mathfrak {E}}_{DHM}$, and ${\mathfrak {E}}_{DF}$ achieves the highest r-value (r=0.982) with F-values of 805.95, 763.71, 763.90, and 762.47, and low standard errors of 12.52, 12.85, 12.85, and 12.86, respectively. This indicates that these entropies have a significant impact on FP, making them the most effective predictors. Other entropies also show notable predictive power.
- The findings show that the model predicting MW using ${\mathfrak {E}}_{DM1}$ achieves the highest r-value (0.96063) and F-value (322.7449), along with the lowest SE (16.03084). This highlights the model’s strong significance, with ${\mathfrak {E}}_{DM1}$ playing a key role in predicting MW. Other entropies, including ${\mathfrak {E}}_{DM3^*}$, ${\mathfrak {E}}_{DM2}$, ${\mathfrak {E}}_{DHM}$, and ${\mathfrak {E}}_{DF}$, also show solid predictive accuracy for MW.
- The analysis shows that the model estimating the impact of entropy ${\mathfrak {E}}_{DM3^*}$ on P and XLogP3 achieves the highest statistical indicators, with strong r-values (0.97087 and 0.9518), F-values (443.2443 and 259.9772), low SEs (1.919 and 0.40563), and a probability of $<0.0001$. These results confirm the model’s robustness, identifying ${\mathfrak {E}}_{DM3^*}$ as the most significant predictor of P and XLogP3. However, the other nine models also demonstrate strong statistical performance, with metrics close to those of the leading model, indicating that other entropies also effectively predict P and XLogP3.
- The findings indicate that the model using ${\mathfrak {E}}_{DM1}$ and ${\mathfrak {E}}_{DM3^*}$ to predict C yields the highest r-value (0.928) and F-value (168.152), along with the lowest SE (40.43). This strong significance underscores the role of ${\mathfrak {E}}_{DM1}$ as a key predictor of C. Additionally, other entropies, such as ${\mathfrak {E}}_{DM2}$, ${\mathfrak {E}}_{DHM}$, and ${\mathfrak {E}}_{DF}$, also demonstrate reliable predictive accuracy for C.

**Figure 4:**
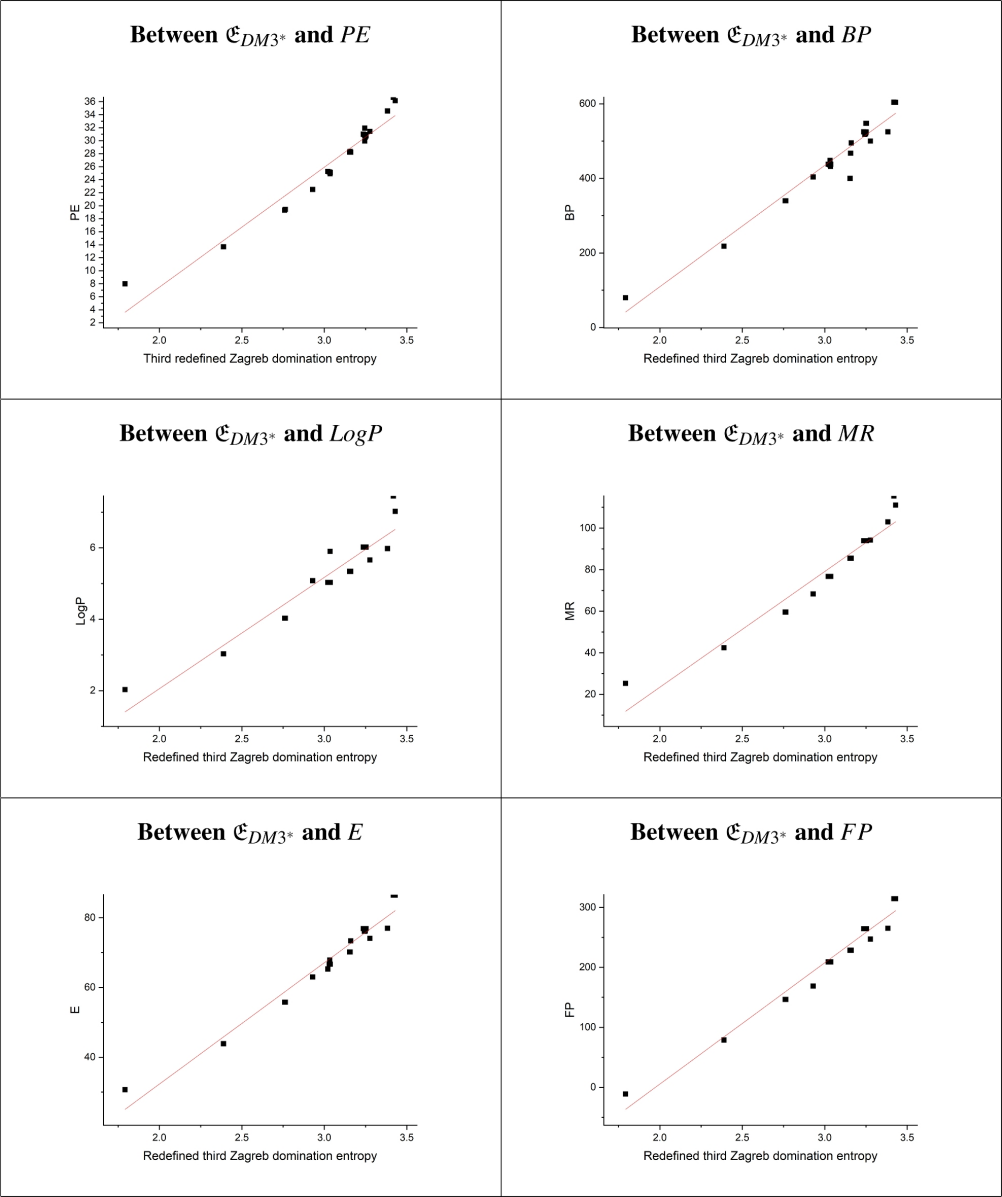
Scatterplots illustrating the linear relationship of entropies vs. PE and physical properties of benzenoid hydrocarbons (Part 1).

**Figure 5:**
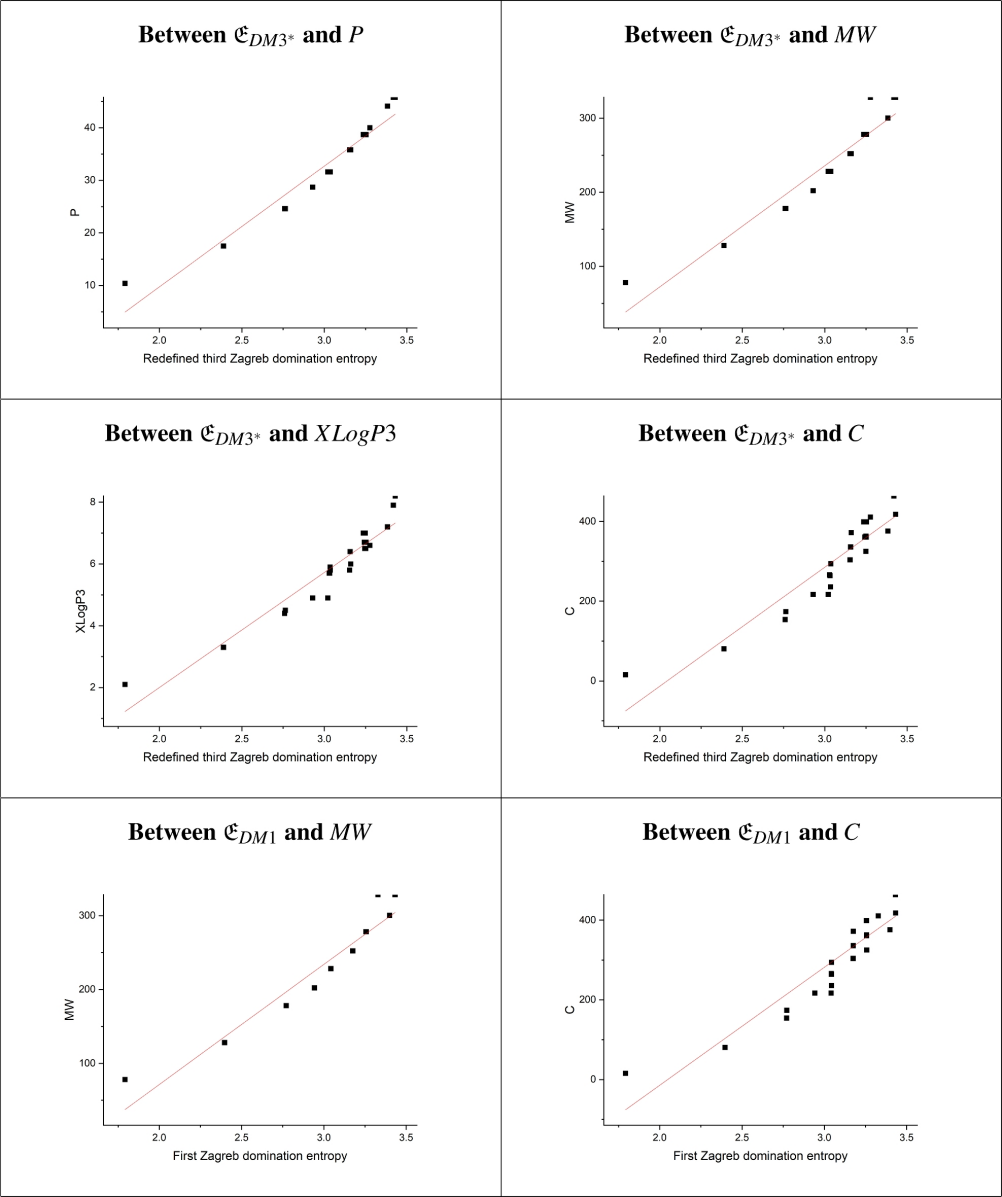
Scatterplots illustrating the linear relationship of entropies vs. physical properties of benzenoid hydrocarbons (Part 2).

In this study, the correlation coefficients between all domination entropies and the physicochemical properties range from 0.92 to 0.98. Based on the observations above, ${\mathfrak {E}}_{DM3^*}$ stands out as the most effective domination entropy for predicting the physicochemical properties and $\pi$-electronic energy of benzenoid hydrocarbons. However, since the r-values for all models exceed 0.92, it indicates that the other entropies also act as strong predictors. Scatterplots demonstrating the linear correlation between the most significant domination entropies and the $\pi$-electronic energy, along with the physicochemical properties of benzenoid hydrocarbons, are presented in Figs. 4 and 5.

In the article^28^, a quality testing analysis of Ve-degree based entropies was performed using 18 benzenoid hydrocarbons, which showed good correlation with certain properties. However, our model provides more accurate predictions of the physicochemical properties of benzenoid hydrocarbons, due to a larger sample size, higher F-value, lower probability, and a linear regression model that exhibits the strongest correlation with the data. Additionally, our study incorporates some other physicochemical properties of benzenoid hydrocarbons that were not included in^28^.

### Multilinear regression analysis

The multilinear regression analysis^30^ employs the following model equation:

$$\begin{aligned} P = \alpha + \beta _1 {\mathfrak {E}}_{DABC} + \beta _2 {\mathfrak {E}}_{DM2} + \beta _3 {\mathfrak {E}}_{DHM} + \beta _4 {\mathfrak {E}}_{DM2^*} + \beta _5 {\mathfrak {E}}_{DM3^*}, \end{aligned}$$

where $P$ represents the physicochemical property or $\pi$-electronic energy, $\beta _i$ ($i = 1, 2, 3, 4, 5$) are the regression coefficients, and $\alpha$ is a constant. The following are the best fitting multilinear regression models for the nine physicochemical properties and $\pi$-electronic energy of benzeneoid hydrocarbons.

$$\begin{aligned} PE&= -27.352 -9050.530 {\mathfrak {E}}_{DABC} -3302.085 {\mathfrak {E}}_{DM2}+3787.245 {\mathfrak {E}}_{DHM}+9564.209 {\mathfrak {E}}_{DM2^*}\\&\quad -980.753 {\mathfrak {E}}_{DM3^*}\\ BP&= -521.042 -185259.551 {\mathfrak {E}}_{DABC} -31727.012 {\mathfrak {E}}_{DM2}+32603.173 {\mathfrak {E}}_{DHM}+201202.717 {\mathfrak {E}}_{DM2^*}\\&\quad -16492.531 {\mathfrak {E}}_{DM3^*}\\ Log P&= -3.938 -1431.901 {\mathfrak {E}}_{DABC} -566.044 {\mathfrak {E}}_{DM2}+647.875 {\mathfrak {E}}_{DHM}+1490.216 {\mathfrak {E}}_{DM2^*} \\&\quad -136.987 {\mathfrak {E}}_{DM3^*}\\ MR&= -82.096 -26672.343 {\mathfrak {E}}_{DABC} -10100.642 {\mathfrak {E}}_{DM2}+11804.768 {\mathfrak {E}}_{DHM}+27912.953 {\mathfrak {E}}_{DM2^*}\\&\quad -2889.680 {\mathfrak {E}}_{DM3^*}\\ E&= -34.882 -13888.283 {\mathfrak {E}}_{DABC} -4707.716 {\mathfrak {E}}_{DM2}+5622.401 {\mathfrak {E}}_{DHM}+14449.713 {\mathfrak {E}}_{DM2^*}\\&\quad -1441.188 {\mathfrak {E}}_{DM3^*}\\ FP&= -386.534 -71397.759 {\mathfrak {E}}_{DABC} -24693.799 {\mathfrak {E}}_{DM2}+30121.395 {\mathfrak {E}}_{DHM}+73813.921 {\mathfrak {E}}_{DM2^*}\\&\quad -7640.819 {\mathfrak {E}}_{DM3^*}\\ P&=-33.79188 -10426.334 {\mathfrak {E}}_{DABC} -3970.269 {\mathfrak {E}}_{DM2}+4575.691 {\mathfrak {E}}_{DHM}+10978.313 {\mathfrak {E}}_{DM2^*}\\&\quad -1134.826 {\mathfrak {E}}_{DM3^*}\\ MW&= -235.626 -81640.102 {\mathfrak {E}}_{DABC} -29580.319 {\mathfrak {E}}_{DM2}+34092.247 {\mathfrak {E}}_{DHM}+86218.749 {\mathfrak {E}}_{DM2^*}\\&\quad -8930.566 {\mathfrak {E}}_{DM3^*}\\ XLog P3&= -5.125 -2016.744 {\mathfrak {E}}_{DABC} -625.914 {\mathfrak {E}}_{DM2}+746.547 {\mathfrak {E}}_{DHM}+2106.255 {\mathfrak {E}}_{DM2^*}\\&\quad -206.393 {\mathfrak {E}}_{DM3^*}\\ C&= -564.903 -183554.239 {\mathfrak {E}}_{DABC} -74395.547 {\mathfrak {E}}_{DM2}+62537.144 {\mathfrak {E}}_{DHM}+207705.562 {\mathfrak {E}}_{DM2^*}\\&\quad -12003.855 {\mathfrak {E}}_{DM3^*}\\ \end{aligned}$$

**Table 6:** Summary of the best predictive fits from multilinear regression analysis.

Properties	r	SE	F	Prob
PE	0.979	1.405	108.469	$<0.0001$
BP	0.991	15.914	267.2703	$<0.0001$
LogP	0.966	0.309	64.857	$<0.0001$
MR	0.976	4.510	96.235	$<0.0001$
E	0.988	1.932	204.203	$<0.0001$
FP	0.991	10.036	254.767	$<0.0001$
P	0.978	1.785	104.158	$<0.0001$
MW	0.966	15.932	66.219	$<0.0001$
XLogP3	0.968	0.358	68.936	$<0.0001$
C	0.945	38.563	38.312	$<0.0001$

Table 6 provides a summary of the statistical parameters for the multiple linear regression models, including the multiple correlation coefficient ($r$), $F$-value, SE, and probability. The analysis evaluates 10 dependent variables PE, BP, LogP, MR, E, FP, P, MW, XLogP3, and C in relation to five predictors: ${\mathfrak {E}}_{DABC}$, ${\mathfrak {E}}_{DM2}$, ${\mathfrak {E}}_{DHM}$, ${\mathfrak {E}}_{DM2^*}$, and ${\mathfrak {E}}_{DM3^*}$. The results in Table 6 indicate that all the multilinear models demonstrate strong predictive capabilities for the physicochemical properties and $\pi$-electronic energy. These models reveal significant multilinear correlations, ranging between 0.945 and 0.991, highlighting the effectiveness of domination entropies in QSPR modeling. Furthermore, the consistently high $F$-values and low probability confirm the statistical significance and robustness of these predictive relationships. The findings underscore the potential of domination entropy-based regression models as reliable tools for predicting key molecular properties. 

## Conclusion

This study demonstrates that domination entropies, derived from domination topological indices, provide valuable insights into the structural and physicochemical properties of benzenoid hydrocarbons. Domination plays a crucial role in understanding the structural attributes of molecular graphs. Ten new domination entropies are introduced, based on domination topological indices, and calculated for 29 benzenoid hydrocarbons. The QSPR between these entropies and the physicochemical properties, as well as the $\pi$-electronic energy, are analyzed. Using linear and multilinear regression models, all regression models are highly significant, with correlation values ranging from 0.92 to 0.99. The entropies effectively predict physicochemical properties and $\pi$-electronic energy, with the redefined third Zagreb domination entropy being the most predictive. These findings highlight the potential of domination entropies as robust tools for predicting molecular properties and guiding further research in molecular design.

However, this study is focused solely on benzenoid hydrocarbons, and its findings may not be applicable to other types of hydrocarbons. It also does not account for environmental factors, such as temperature or solvent effects. Future research could expand the dataset to include a broader range of both benzenoid and non-benzenoid hydrocarbons, as well as higher polycyclic aromatic hydrocarbons, to gain a more comprehensive understanding of structure-property relationships. Moreover, incorporating advanced statistical models, such as machine learning techniques, could uncover complex non-linear interactions overlooked by traditional regression models, enhancing prediction accuracy and providing deeper insights into molecular behavior.

## Data Availability

The datasets generated and/or analyzed during the current study are available in the ChemSpider (https://www.chemspider.com/) and PubChem (https://pubchem.ncbi.nlm.nih.gov/) repositories. The chemical structures of the drugs were obtained from ChemSpider (https://www.chemspider.com/). The research utilized OriginPro software, which is available at https://www.originlab.com/.

## Code availability

The domination entropy calculations are verified using Python, and the code is available at https://doi.org/10.5281/zenodo.15030004.
